# Prognostic marker VPS72 could promote the malignant progression of prostate cancer

**DOI:** 10.1186/s12885-024-12488-z

**Published:** 2024-06-10

**Authors:** Xiaolong Xu, Wei Wang, Yi He, Yiqun Yao, Bo Yang

**Affiliations:** 1 Department of Urology, the Second Affiliated Hospital of Dalian Medical University, 467, Zhongshan Road, Shahekou District, Dalian, Liaoning 116001 China; 2 Department of Breast and Thyroid Surgery, the Affiliated Zhongshan Hospital of Dalian University, 6, Jiefang Street, Zhongshan District, Dalian, Liaoning 116001 China

**Keywords:** Prostate cancer, VPS72, Clinical prognosis, Nomogram

## Abstract

**Background:**

This paper attempted to clarify the role and mechanism of vacuolar protein sorting-associated protein 72 homolog (VPS72) in the progression of prostate cancer (PCa).

**Methods:**

Clinical information and gene expression profiles of patients with prostate cancer were obtained from The Cancer Genome Atlas (TCGA). VPS72 expression in PCa and the potential mechanism by which VPS72 affects PCa progression was investigated. Next, we performed COX regression analysis to identify the independent prognostic factors of PCa, and constructed a nomogram. The sensitivity of chemotherapeutic medications was anticipated using “pRRophetic”. Subsequently, in vitro assays to validate the effect of VPS72 on PCa cell proliferation, migration and susceptibility to anti-androgen therapy.

**Results:**

The expression of VPS72 was considerably higher in PCa tissues compared to normal tissues. Significant correlations were found between high VPS72 expression and a poor prognosis and adverse clinicopathological factors. The nomogram model constructed based on VPS72 expression has good predictive performance. According to GSEA, VPS72-related genes were enriched in the NF-kB pathways, cytokine-cytokine receptor interaction and chemokine signaling pathway in PCa. Although PCa with low VPS72 expression was more adaptable to chemotherapeutic medications, our in vitro experiment showed that VPS72 knockdown significantly decreased the PCa cell migration, proliferation, and resistance to anti-androgen therapy.

**Conclusions:**

In summary our findings suggests that VPS72 could play a crucial role in the malignant progression of PCa, and its expression level can be employed as a possible biomarker of PCa prognosis.

## Introduction

Globally, prostate cancer (PCa) is the fifth most common cause of cancer-related mortality and the second most common cancer in men [1]. In China, the incidence of prostate cancer and cancer-related mortality started to rise in 2012 due to the introduction of prostate-specific antigen (PSA) screening and the increasing rate of population aging [2]. Patients with locally advanced and metastatic PCa often benefit from the combination of androgen deprivation therapy (ADT) and androgen blocking during the initial phase of treatment. However, because of its heterogeneity, PCa has a broad illness spectrum that includes subtypes that are clinically indolent as well as aggressive ones. Only 5–10% of individuals survive ten years after starting ADT for hormone-sensitive prostate cancers (HSPC), which almost always develop to castration-resistant prostate cancers (CRPC) within five years [3]. Multiple randomized controlled phase-III trials, including CHAARTED [4] and LATITUDE [5], demonstrated that when HSPC patients are found to have either a long- or short-term progression to CRPC before initiating treatment, it is possible to implement an early and appropriate follow-up strategy. Finding relevant and successful therapy targets is extremely difficult due to high heterogeneity, complex makeup, and etiology. Therefore, in order to address the demanding clinical needs, novel biomarkers and therapeutic techniques are desperately needed.

Vacuolar protein sorting (VPS) plays a critical role in the production and secretion, exocytosis, endocytosis, and recovery of intracellular molecules by decarbonizing them [6]. The GeneCards database, available at www.genecards.org, contains 30 human VPS genes. The relationship between human diseases and the genes and products of VPSs is a topic of limited research. As a part of the SRCAP complex, vacuolar protein sorting-associated protein 72 homolog (VPS72) is primarily found in the nucleus. It mediates the exchange of the ATP-dependent histone H2AZ1/H2B dimer with nucleosome H2A/H2B, which in turn causes chromatin remodeling, which in turn regulates transcription of specific genes [7]. Few publications have shown that VPS72/YL1-mediated H2A.Z deposition is an essential step for nuclear recombination to take place following mitosis [8]. Until now, substantially less is known about VPS72 in cancer. Chen et al. revealed that VPS72 knockdown inhibited the growth and migration of HCC cells in vitro and inhibited the AKT signaling pathway [9]. Consistently, Zhang et al. recently demonstrated that VPS72 could enhance the affinity of MYC for its target gene promoters and promoted their transcription, thereby contributing to HCC progression [10]. However, the roles of VPS72 in PCa remains unknown.

This study investigated expression profiles, prognostic implications and potential function of VPS72 in PCa, and confirmed the effect of VPS72 on the in vitro migration, proliferation, and the sensitivity to anti-androgen therapy in LNCaP cells. Our results suggest that VPS72 could be a therapeutic target for PCa and provide fresh information on the function of VPS72 in the malignant progression of PCa.

## Materials and methods

### Data extraction

The RNA-seq data spanning 33 tumor types and normal tissues of 15,776 samples were obtained from The Cancer Genome Atlas (TCGA) database and the Genotype-Tissue Expression (GTEx) database by UCSC XENA in order to completely understand the expression of the VPS72 gene in common human malignancies. From the expression profiles of these tumors, we retrieved the VPS72 gene expression data. We next compared the clinical data of the individuals with the corresponding gene expression data.

### Analysis of VPS72 expression in PCa

The R language “limma” package was used to extract the VPS72 expression data in PCa, while the “pROC” program was used for diagnostic analysis and differential expression analysis as well as visualization. The associated patient prognosis data and the gene expression data were then combined, and prognostic measures like the progression-free interval (PFI) were examined.

### Prognostic analysis of VPS72 expression in PCa

Univariate and multivariate Cox regression analysis were used to determine the predictive values of VPS72 in PFI of PCa patients in order to ascertain whether the impact of VPS72 expression on the prognosis of patients with PCa is independent of other clinical variables.

### DEGs between VPS72 high expression and low expression groups in PCa

Using the deseq2 program, we investigated the DEGs between various VPS72 expression groups in PCa (low expression group: 0–50%; high expression group: 50–100%). Using the threshold parameters of |log2 fold-change (FC)| > 1.0 and adjusted *p*-value < 0.05, the ggplot2 software created the volcano map. Then, using the cluster Profiler statistical analysis software and the ggplot2 visualization package, we carried out KEGG and GO enrichment analyses of DEGs.

### Gene set enrichment analysis (GSEA)

In order to ascertain the contribution of each gene to the phenotype, the trend in the distribution of a specified gene set ranked according to its connection with the phenotype can be evaluated using the useful technique known as GSEA. The VPS72 gene expression was discovered to be associated with relevant gene pathways, which were identified by GSEA. Gene set permutation was carried out 1000 times for every analysis. Gene sets were deemed considerably enriched if their normal *p*-value was less than 5% and their false discovery rate (FDR) was less than 25%.

### Evaluation of chemotherapeutic medication sensitivity

The sensitivity of chemotherapeutic medications for the treatment of PCa was evaluated using the R package “pRRophetic,” and the sensitivity of each medication was compared based on VPS72 expression. The half-maximal inhibitory concentration (IC50) was an indicator of the response rate of chemotherapeutic drugs to tumor cells. A threshold of *p* < 0.001 was established for statistical significance. Box plots were utilized to show the anticipated sensitivity of each drug using the R package “ggplot2.”.

### Cell culture and transfections

The American Type Culture Collection (ATCC) provided the androgen-sensitive LNCaP prostate cancer cells, which were then cultured in RPMI-140 (Invitrogen, Carlsbad, CA) supplemented with 10% fetal bovine serum (Hyclone, USA), Penicillin-Streptomycin (100 U/ml), and glutamine (2 mM, Hyclone) at 37 °C in a humidified environment with 5% CO2. After being separated by 0.25% trypsin and 0.02% EDTA solution, the cells were subcultured once every two to three days. In order to create 3D cultures, cells were cultured in a gel mixture that contained 0.05 M HEPES, 3.7 g/L NaHCO3, 0.5 mg/ml collagen (Corning, Lot# 5,092,001), and 6 mg/ml matrigel (Corning, Lot# 5,061,003).

A lentiviral shRNA vector targeting VPS72 was generated by inserting stranded oligonucleotides (shVPS72, forward sequence 5′- CCG GGG ATG AAC CAT CCA GTG ATG GCT CGA GCC ATC ACT GGA TGG TTC ATC CTT TTT G-3′) into TRC2-pLKO-puro Vector (Sigma-Aldrich). Cells were infected with the VPS72 shRNA vectors and selected with puromycin (5 µg/mL). The transfection efficiency of VPS72 was confirmed by quantitative real-time PCR analysis.

### Flow cytometry analysis of the cell cycle

After cell harvesting, 6-well plates were planted. Following a 24-hour incubation period, the cells were digested, collected, and centrifuged for five minutes at 4 °C at 1000 rpm. After being suspended in ice-cold PBS and cleaned, the deposited cells were again suspended in 70% ethanol for 30 min at 4 °C. After that, the cells were cleaned and resuspended in 100 milliliters of PBS that had been mixed with 0.25% Triton X-100 and 50 mg/mL of RNase A (Sigma, US) for 30 min at 4 °C. In the end, the cells were stained for 30 min with 10 mg/mL propidium iodide (PI; lot: 1,685,935, Life Technologies, USA). The cells were then examined right away in a Becton Dickinson, CA, USA, FACScan flow cytometer.

### Cell apoptosis analysis

Six-well plates were used to seed cells at a density of 1 × 10^5^ cells/well. There were two milliliters of culture media in each well. After a 24-hour incubation period, the cells were subjected to a 24-hour exposure to 10 µM Bicalutamide (Beyotime Biotechnology). After that, the cells were gathered, cleaned with PBS, and then put back into 300 µl of binding buffer. Subsequently, the cell suspensions were incubated for 15 min in the dark after 5 µl of PI was added. Next, a BD FACSVerse flow cytometer was used to identify the cell apoptotic event.

### Quantitative real-time PCR

Using the RNA isoPlus® Reagent Kit (Takara Biotechnology) and following the manufacturer’s instructions, RNA was extracted from LNCaP cells. Reverse transcription of RNA into cDNA was performed using the PrimeScript® RT Reagent Kit (Takara, Shiga, Japan). Using the SYBR® Premix Ex TaqTM Kit and the 7500 Real-Time PCR System (Applied Biosystems, 7500 Real Time PCR System, Thermo, US), the cDNA was amplified. The following were the cycling conditions: 40 cycles of 30 s at 95 °C, 5 s at 95 °C, and 34 s at 60 °C. The results were analyzed using the comparative ΔΔCt technique, with GAPDH serving as the loading control for the target genes. The primers were as follows: VPS72 (Forward: 5’-GAG GAA TGG TTC CCC CAA GG-3’, reverse: 5’-TCC CAA CCC TAG ACT GGA CA-3’; GAPDH (Forward: 5′-GCA CCG TCA AGG CTG AGA AC-3′, reverse: 5′-TGG TGA AGA CGC CAG TGGA-3′).

### Cell proliferation assay

96 well plates were used to seed cells (2 × 105 cells / well). After incubation at 37 °C and 5% CO2 for different times, the CCK-8 kit provided by Tiangen (Hangzhou, China) was mixed at 10 µ L / well, and cells were incubated for 3 h at 37 ° C and 5% CO2. Finally, we read the absorbance at 450 nm on the microplate reader (Thermo Fisher Scientific, Inc.)

### Statistical analyses

SPSS v24.0 (SPSS Inc., USA) or R software (version 3.6.3) were used for all statistical analyses in this work. The Kaplan-Meier survival analysis was conducted using the log-rank test. In the regression analysis, 95% confidence intervals (CIs) and hazardous ratios (HRs) were computed. Using the “pROC” and “timeROC” packages, ROC curves and time-dependent ROC curves were generated to determine the diagnostic value of MYBL2. For group comparisons, the Kruskal-Wallis test and the Student’s t test were employed. A statistically significant value was defined as a two-tailed *P* value less than 0.05. The association between VPS72 protein expression and the clinicopathological characteristics of PCa was examined using the chi-square test or Fisher’s exact test.

## Results

### Transcription levels of VPS72 in PCa and normal tissues

**Fig. 1:**
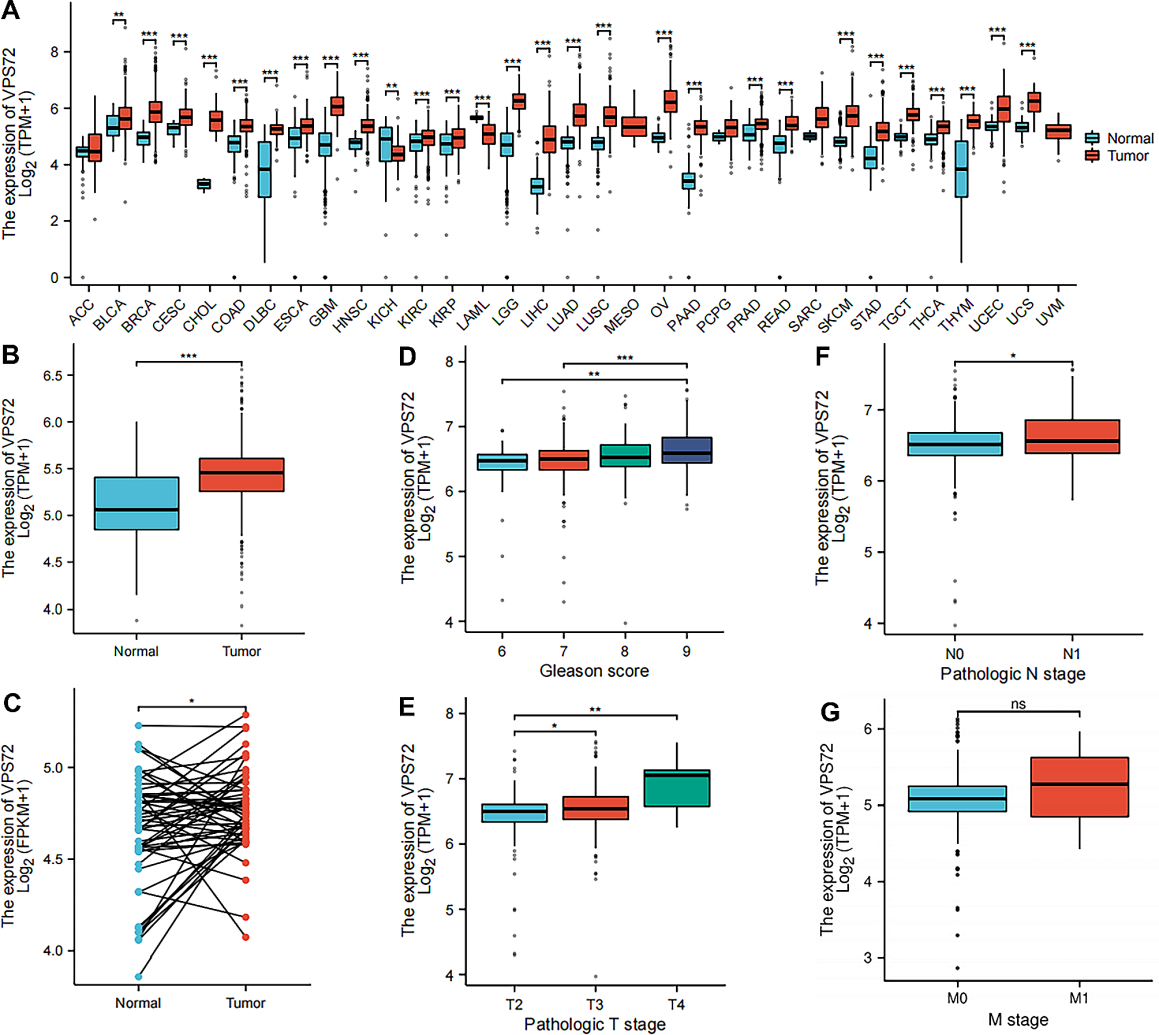
The VPS72 expression pattern and its correlation with the clinical features of PCa patients. (**A**) Expression of VPS72 in pan-cancer samples and normal tissues. (**B**) Expression of VPS72 in PCa and normal tissues. (**C**) Expression of VPS72 in PCa and a matching, nearby normal sample. Relationship between VPS72 expression level and the (**D**) Gleason Score; (**E**) T stage; (**F**) N stage; (**G**) M stage. (ns *p* ≥ 0.05, *P < 0.05; **P < 0.01; ***P < 0.001)

Our understanding of the overall expression of the gene in human cancers has been greatly enhanced by pan-cancer investigation of VPS72 expression. Thus, the VPS72 expression data from the TCGA and GTEx databases was examined first. The outcomes demonstrated that. It was downregulated in KICH and LUSC, but considerably increased in 26 different human cancer types, including BLCA, BRCA, CESC, CHOL, COAD, DLBC, ESCA, GBM, HNSC, KIRC, KIRP, LGG, LIHC, LUAD, LUSC, OV, PAAD, PRAD, PEAD, SKCM, STAD, TGCT, THCA, UCEC, and UCS (Fig. 1A). This shows that the VPS72 gene may function as an oncogene and contribute to the occurrence and growth of cancers. Analysis of unpaired expression data revealed that PCa tissues had considerably greater VPS72 mRNA expression levels than those in normal tissues (Fig. 1B). Then, we analyzed 52 pairs of PCa tissues obtained from the TCGA databases. The level of VPS72 was markedly higher in PCa tissues than in adjacent normal tissues (Fig. 1C).

### Relationship between VPS72 expression levels and PCa clinicopathological parameters

A total of 499 patients with complete clinical and survival data were obtained from the TCGA database in order to further investigate the relationship between VPS72 expression level and PCa clinical characteristics. The patients were then categorized into high and low VPS72 expression groups according to the median expression value of VPS72. Table 1 shows that a number of clinicopathological features of PCa, such as Pathologic T stage (*p* = 0.040), Pathologic N stage (*p* = 0.029), and Gleason score (*p* = 0.048), were strongly correlated with the expression level of VPS72. Higher VPS72 expression levels were seen in patients with high T stage (*p* < 0.001), high Gleason score (*p* < 0.05), and high N stage (*p* < 0.05), as Fig. 1D-G demonstrates. The expression levels of VPS72 did not, however, show any statistically significant link with other clinical pathological parameters, such as age (*p* = 0.137), Clinical M stage (*p* = 1.000), primary therapeutic outcome (*p* = 0.209), and residual tumor (*p* = 0.331). When combined, these findings suggested that a patient’s poor prognosis from PCa may be predicted by a high expression of VPS72.

**Table 1:** Correlations between the VPS72 expression levels and clinicopathologic characteristics in PCa

Characteristics	Low expression of VPS72	High expression of VPS72	*P* value
*N*	250	251	
Age, *n* (%)			0.137
<= 60	104 (20.8%)	121 (24.2%)	
> 60	146 (29.1%)	130 (25.9%)	
Pathologic T stage, *n* (%)			0.040
T2	106 (21.5%)	83 (16.8%)	
T3	137 (27.7%)	157 (31.8%)	
T4	3 (0.6%)	8 (1.6%)	
Pathologic N stage, *n* (%)			0.029
N0	182 (42.5%)	166 (38.8%)	
N1	31 (7.2%)	49 (11.4%)	
Clinical M stage, *n* (%)			1.000
M0	227 (49.3%)	230 (50%)	
M1	1 (0.2%)	2 (0.4%)	
Primary therapy outcome, *n* (%)			0.209
PD	13 (3%)	16 (3.6%)	
SD	20 (4.5%)	10 (2.3%)	
PR	17 (3.9%)	23 (5.2%)	
CR	173 (39.3%)	168 (38.2%)	
Gleason score, *n* (%)			0.048
6	28 (5.6%)	18 (3.6%)	
7	134 (26.7%)	114 (22.8%)	
8	31 (6.2%)	34 (6.8%)	
9	55 (11%)	83 (16.6%)	
10	2 (0.4%)	2 (0.4%)	
Residual tumor, *n* (%)			0.331
R0	164 (34.9%)	152 (32.3%)	
R1	72 (15.3%)	77 (16.4%)	
R2	4 (0.9%)	1 (0.2%)	

Abbreviations: PD: Progressive disease; SD: Stable disease; PR: Partial Response; CR: complete response; R0: No residual tumor; R1: Microscopic residual tumor; R2: Macroscopic residual tumor

### Prognostic analysis of VPS72 expression levels in PCa

**Fig. 2:**
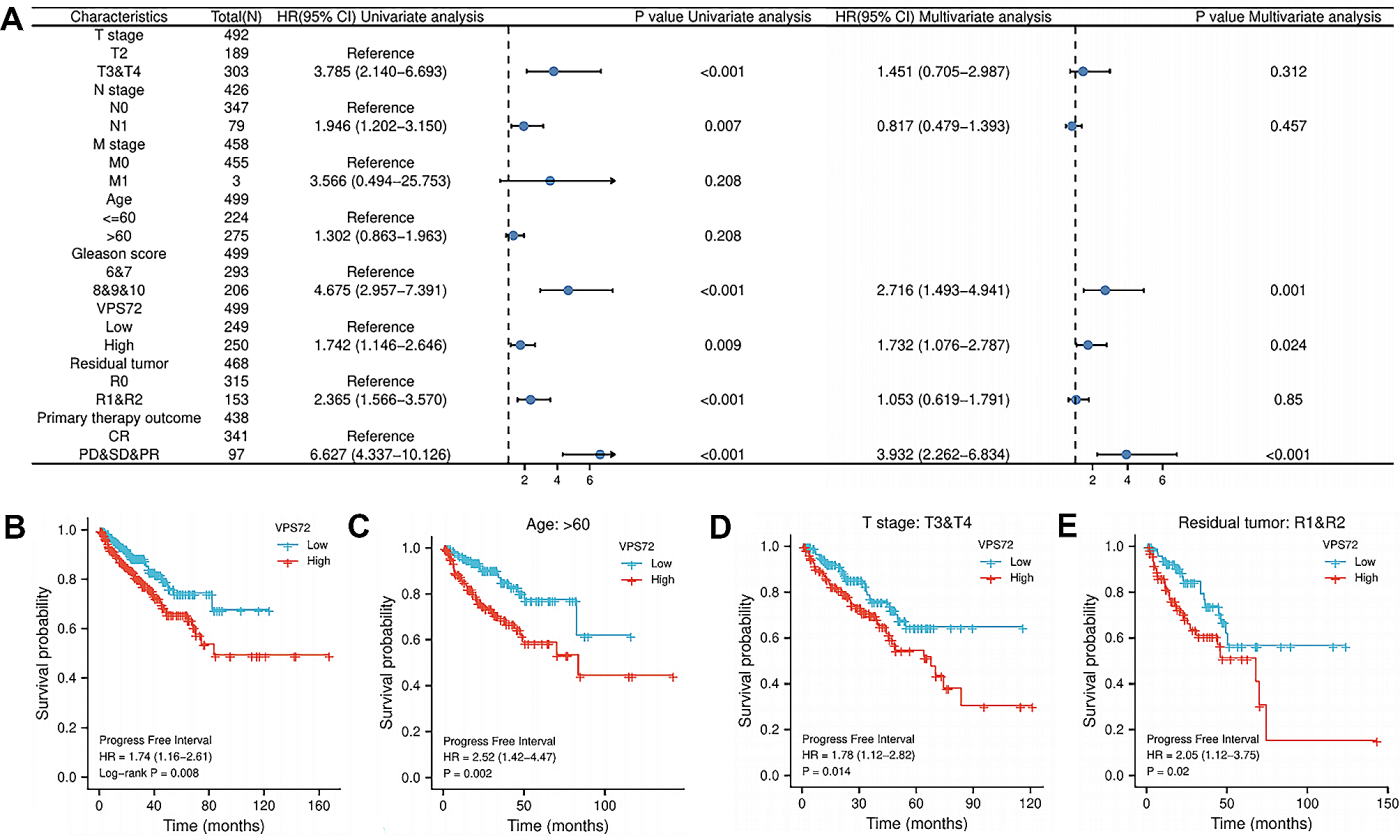
Prognostic value of VPS72 expression level in PCa that is independent. (**A**) The findings of the univariate and multivariate Cox regression analyses are displayed in the forest plot. (**B**) Progression-free interval (PFI) for VPS72 in all patients was analyzed using the Kaplan-Meier curve; (C–E) age, residual tumor, and T stage-specific Kapla-Meier survival analyses were performed

To find out more about whether VPS72 expression may be employed as a stand-alone prognostic factor, we performed a Cox regression analysis. The Cox survival analysis for PFI revealed a significant prognostic difference on both univariate (HR 1.742, 95% confidence interval [CI]: 1.146–2.646, *p* = 0.009) and multivariate (HR 1.732, 95%CI: 1.076–2.787, *p* = 0.024) comparisons between the high and low VPS72 expression groups (Fig. 2A). As seen in Fig. 2B, the Kaplan-Meier survival analysis revealed a significant correlation between increased VPS72 expression and shorter PFI (*p* = 0.008). High VPS72 expression was substantially correlated with a poor prognosis in PCa cases aged > 60 (*p* = 0.008), T stage: T3&T4 (*p* = 0.014), and residual tumor: R1&R2 (*p* = 0.02), according to a stratified analysis by distinct individual clinical characteristics, as seen in Fig. 2C–E.

### Diagnostic value of VPS72 expression levels in PCa

**Fig. 3:**
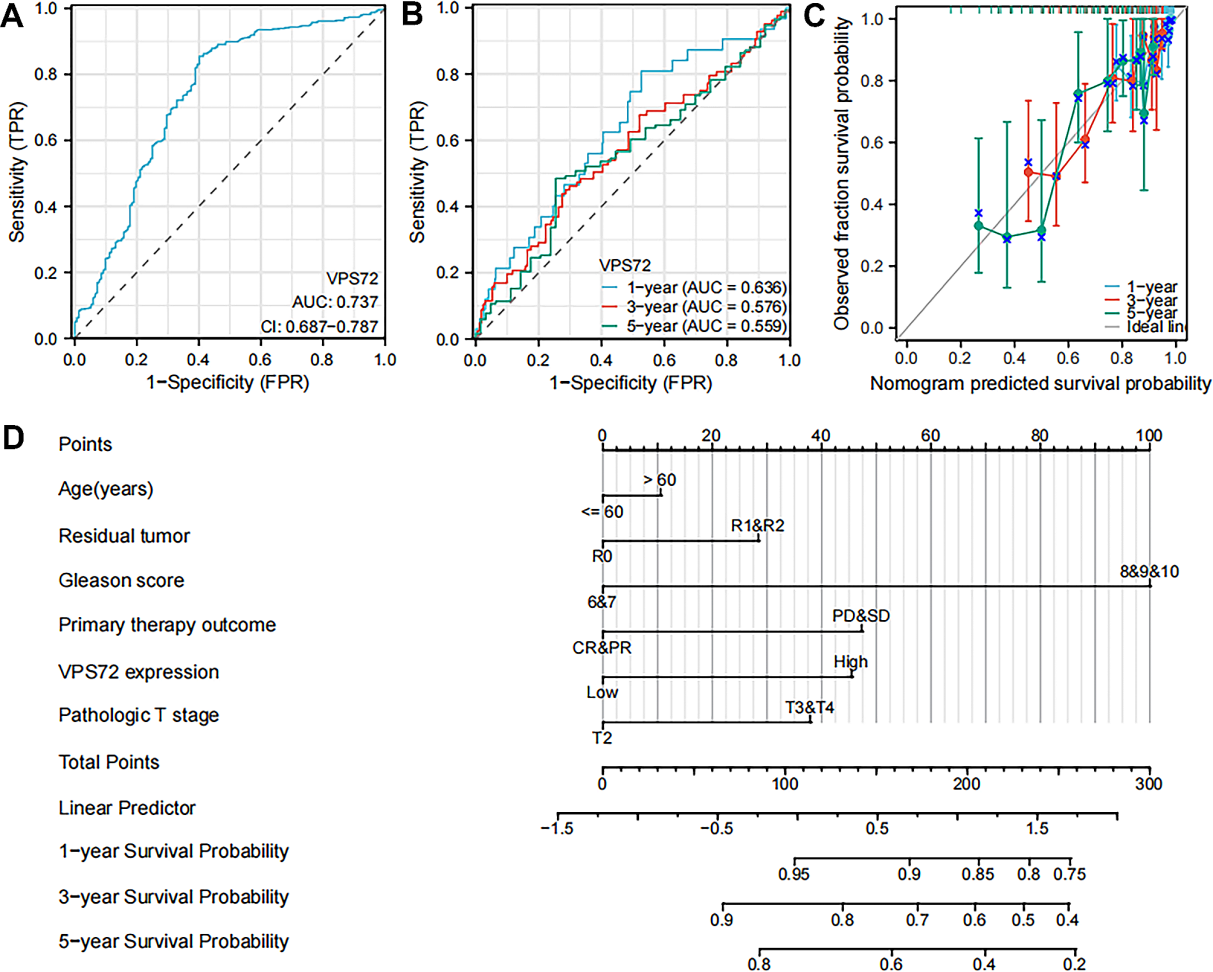
VPS72 expression has diagnostic relevance for PCa patients. (**A**) The VPS72 ROC curve in PCa and normal prostate tissue. (**B**) The VPS72 time-dependent ROC curve in both PCa and normal prostate tissue. (**C**) Calibration plots showing the degree of agreement between the real survival rate and the rate predicted by the nomogram. (**D**) Nomogram for estimating the likelihood that PCa patients with 1, 3, and 5-year PFI

After that, the diagnostic efficacy of VPS72 expression for PCa was assessed using ROC curves. The ROC curve for VPS72 indicated an AUC value of 0.737 (95% CI: 0.687–0.787), as seen in Fig. 3A. VPS72’s sensitivity and specificity were 85.4% and 59.8%, respectively, at a cutoff of 5.1795. These findings suggested that VPS72 might be a useful biomarker for distinguishing PCa tissues from healthy tissues. Additionally, we generated time-dependent ROC curves and discovered that VPS72’s AUC value performed reasonably well initially before declining over time (Fig. 3B). Nomograms are frequently used to forecast cancer patients’ prognoses. The calibration and discriminating power of nomograms are frequently examined using the C-index and calibration plots. In this study, by integrating the expression level of VPS72 with clinical factors, we created a nomogram based on the findings of the multivariate Cox proportional hazards analysis to predict the 1-, 3-, and 5-year survival probability of patients. The calibration plot showed that the 1-year, 3-year, and 5-year PFI probabilities predicted by our nomogram were congruent with the actual results. The nomogram’s C-index was 0.730 (Fig. 3C). For tailored clinical assessments and treatment plans, our nomogram may offer useful information (Fig. 3D).

### Biological function of VPS72 in PCa

**Fig. 4:**
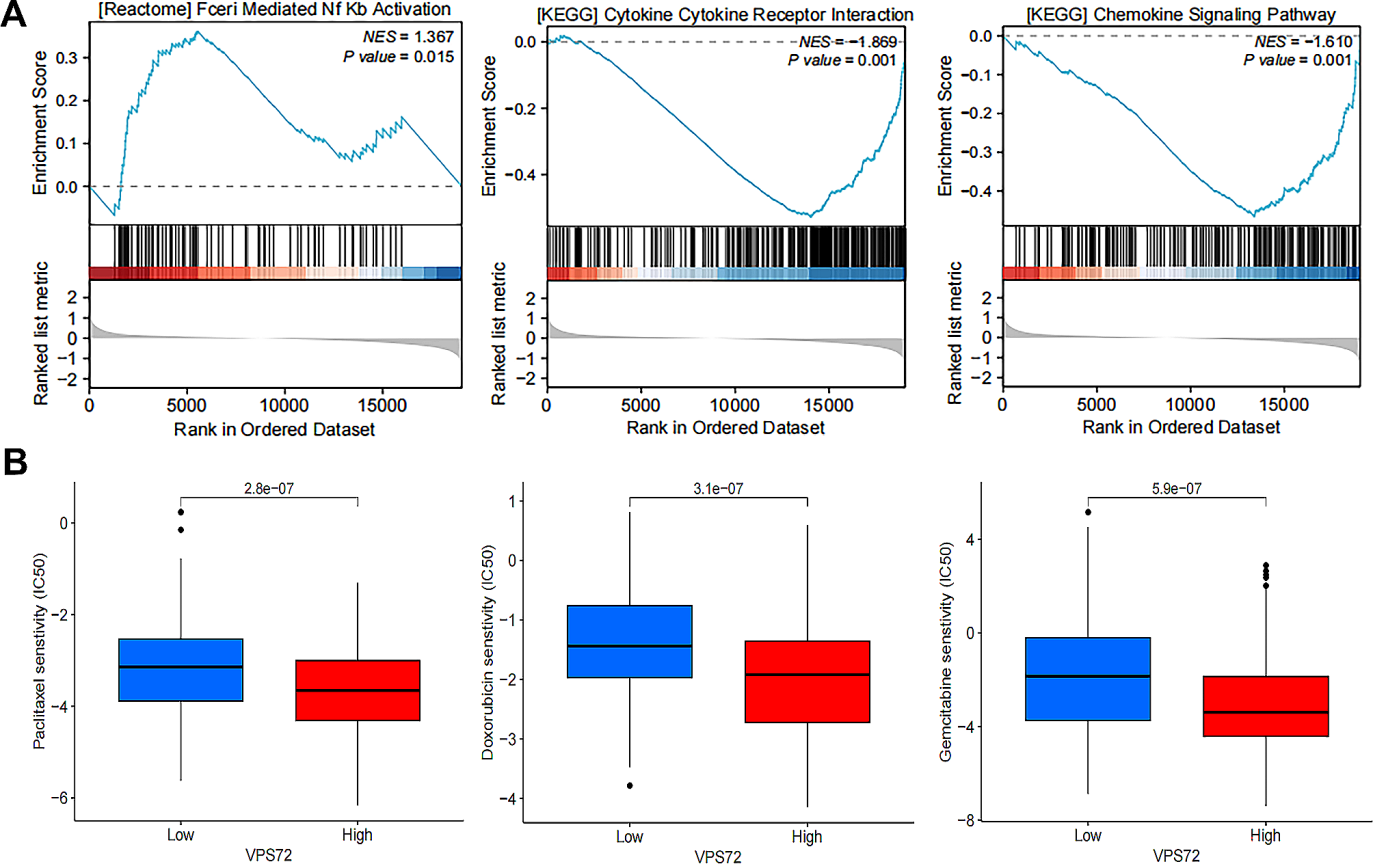
Biological Function of VPS72 expression levels in PCa. (**A**) GSEA pathway enrichment analysis between patients with high and low VPS72 expression. (**B**) Correlations of VPS72 expression with the sensitivity of chemotherapeutic medications in PCa

Functional enrichment analysis was used to examine the transcriptome data from the TCGA database in order to comprehend the possible molecular mechanism underlying the prognostic significance of VPS72 in PCa. Groups with VPS72-high and VPS72-low expression levels were created based on the PCa samples’ median expression levels. To determine which signaling pathways were differently activated, we performed GSEA compared groups with high and low VPS72 expression. According to GSEA data, patients exhibiting high expression levels of VPS72 may have active NF-kB pathways while potentially suppressing the cytokine-cytokine receptor interaction and chemokine signaling pathway (Fig. 4A). Next, the IC50 scores of different chemotherapy and targeted drugs in the low and high VPS72 expression groups were predicted. Interestingly, the result indicated that compared with the high expression group, the low expression group had higher IC50 scores of Paclitaxel, Doxorubicin and Gemcitabine (Fig. 4B). Considering that AR was the most well-characterized driver of PCa, further meticulous wet lab studies were required.

### The oncogenic roles of VPS72 in PCa LNCaP cells

**Fig. 5:**
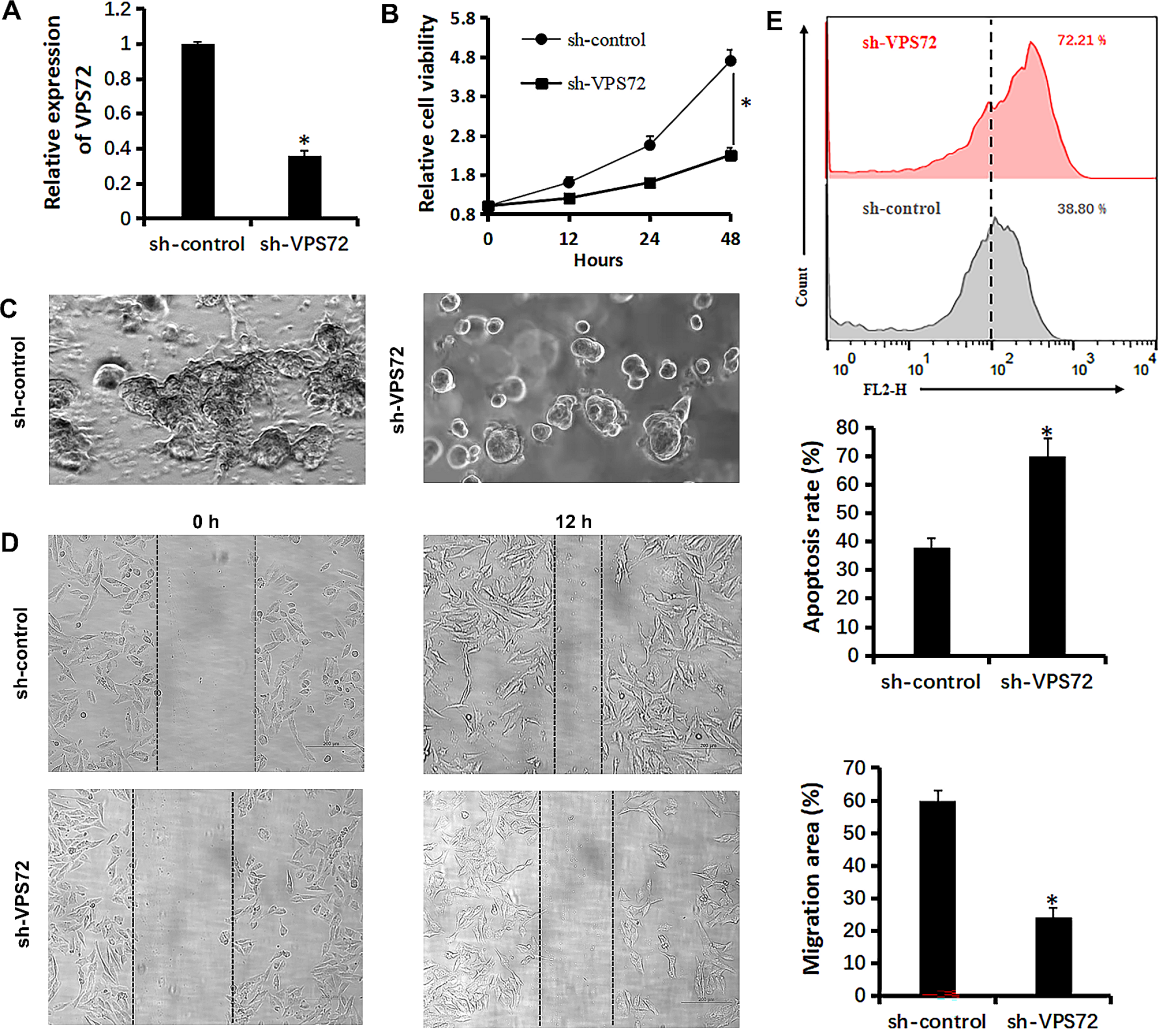
Relationship between VPS72 and viability of LNCaP cells. (**A**) qRT-PCR assay shows the transcriptional levels of the VPS72 gene with GAPDH used as the loading control in LNCaP cells. (**B**) Effect of sh-VPS72 on the proliferation of LNCaP cells was detected by CCK-8 assays. (**C**) Changs in cellular invasiveness of LNCaP cells were investigated via 3D culture. (**D**) Migration of LNCaP cells was detected via wound healing assay. (**E**) Apoptosis of LNCaP cells induced by Bicalutamide (10µM) were measured by flow cytometry analyses. Data are presented as the mean ± SD for three independent experiments (*P < 0.05)

Therefore, the shRNA vector was used to specifically and steadily lower VPS72 expression in LNCaP cells in order to evaluate the functions of VPS72 in PCa. Expression levels were measured by quantitative real-time PCR (Fig. 5A). In 3D preparations of LNCaP cells, reduced VPS72 expression impeded invasiveness and reduced cell viability (Fig. 5B). Furthermore, LNCaP cells’ ability to migrate was hindered by decreased VPS72 expression (Fig. 5C). Flow cytometry results demonstrated a considerable increase in sensitivity to Bicalutamide (10 µM) therapy upon VPS72 knockdown (Fig. 5D).

## Discussion

Prostate cancer, which has a comparatively high death rate, is the second most prevalent cancer diagnosed globally. Male cancer-specific mortality has been thought to be mostly caused by PCa in recent decades [11]. While over 80% of patients with primary PCa survive for five years or longer, advanced PCa always progressed two to three years after initial therapy and develops resistance to ADT [12]. These cases ultimately culminate in CRPC, for which there is now no viable treatment [13]. Although the prognosis of PCa have been dramatically improved by standardized surgical treatments, the clinical staging of newly diagnosed PCa patients in China differs from western developed countries. For instance, among the newly diagnosed PCa patients in the US, most cases (~ 76%) are clinically localized, while only ~ 13% and ~ 6% involve metastases in local lymph nodes or distant sites, respectively [14]. However, the data in China are quite different. A multi-center Chinese study showed that only 1/3 of the newly diagnosed PCa patients are clinically localized. Also, most patients in China are in the middle or advanced stage at the diagnosis, resulting in a worse overall prognosis than in western countries [15]. Thus, new biomarkers and therapeutic approaches are urgently needed to meet the challenging clinical needs.

Genetic prognostic markers are essential to the diagnosis and prognosis of cancers, and identifying more prognostic markers can improve our understanding of PCa. Membrane-wrapped vesicles in eukaryotic cells play a role in intracellular molecular transport along organelles such as the Golgi apparatus, endosome, secretory vesicles, lysosomes, and endoplasmic reticulum [16]. There is only a small amount of research on the association between VPSs genes and their products and human diseases. Abnormal VPS expression were found to affect different physiological processes in the human body, including the ubiquitin disruption follows VPS11/VPS18 overexpression [17]. Research pertaining to tumors has also revealed aberrant VPS expression and prognostic values. VPS52 is a tumor suppressor gene in gastric cancer, and its lack is associated with a worse prognosis. Overexpression of VPS52 prevents the growth of tumors in vivo and in vitro [18]. Wang et al. found that the downregulation of VPS33b is a critical stage in the development of inflammation-driven hepatocellular carcinoma (HCC), indicating that it is a critical tumor suppressor in hepatocellular carcinogenesis [19]. However, the role of the human VPS gene and its products in malignancies is largely unknown. Vacuolar protein sorting-associated protein 72 homolog (VPS72), also referred to as YL1 or Swc2, is primarily found in the nucleus as a part of the SRCAP complex. It facilitates the exchange of the ATP-dependent histone H2AZ1/H2B dimer with the nucleosome H2A/H2B, which in turn causes chromatin remodeling to regulate transcription of specific genes [7]. Up to now, much less is known about VPS72, and only a few articles have reported that VPS72/YL1-mediated H2A.Z deposition is a necessary process for nuclear recombination to occur after mitosis [20]. According to research by Chen et al., VPS72 binding to lysine acetyltransferase 5 (KAT5) stimulates HCC cells growth, invasion, and migration by controlling the PI3K/AKT signaling pathway [9]. Nevertheless, there haven’t been any reports on VPS72’s prognostic significance in cancer.

In the current study, we first used extensive database resources and statistical analysis to comprehensively and systematically demonstrate that VPS72 was a poor prognostic factor in the malignant progression of PCa. We evaluated the expression level of VPS72 in pan-cancer by looking through the TCGA and GTEx databases, and discovered that VPS72 was differentially expressed at high levels in the majority of tumor types, but only at low levels in KICH and LUSC. These results indicated that VPS72 could function as a gene that promotes cancer in most malignant tumors and may have important roles in the development of cancer. We further found that there is a favorable correlation between high T stage, high Gleason score, and high N stage and the increased mRNA expression of VPS72. But we also observed that there isn’t a discernible difference between PCa’s M stage and VPS72 expression. The small sample size of patients in the M1 stage may be the cause of this weak connection. Based on Kaplan-Meier curves and univariate Cox regression analysis, we verified that a shorter PFI is linked to increased VPS72 expression. According to multivariate Cox proportional hazards analysis and ROC curve analysis, VPS72 may prove to be a useful diagnostic biomarker for distinguishing early-stage PCa from normal tissues and serving as a stand-alone indicator of a bad prognosis for PCa. Therefore, we developed a nomogram based on the expression of VPS72 that was able to predict PCa patients’ PFI with accuracy.

The results of GSEA may provide some clues on the mechanism by which VPS72 influences the survival in PCa. Interestingly, the results in our study demonstrated that several important signaling pathways, including NF-kB Activation, Cytokine Cytokine Receptor Interaction, and Chemokine Signaling Pathway, were altered between the high and low VPS72 expression groups. Even though the bioinformatic analysis verified that VPS72 promotes malignancy in PCa, in the examination of medication sensitivity, the samples from the high VPS72 expression showed greater sensitivity to the chemotherapy drugs, which have been proven to have therapeutic effects on PCa through clinical trials [21–23]. Taking into consideration that AR was the PCa driver with the best characterization, meticulous in vitro research is needed to corroborate our findings and broaden their therapeutic applicability. We thus further looked into the VPS72 function in LNCaP cells. All things considered, our findings demonstrated that VPS72 knockdown could significantly decrease the cell viability and migration. Importantly, VPS72 knockdown significantly enhanced the sensitivity to the AR antagonists Bicalutamide. These results indicated that the molecular mechanism of VPS72 promoting malignant progression of PCa could be related to the AR signaling pathway.

Our study has a few limitations, despite the fact that it indicated a possible function for VPS72 in the prognosis of PCa. In the absence of real clinical data, we set out to investigate VPS72 exclusively utilizing the public databases. However, biological experiments should be conducted further and provide high-quality proof in the form of precise verification. Therefore, further real-world PCa samples and meticulous wet lab studies were required to investigate the function of VPS72 in PCa.

In conclusion, the current investigation showed that VPS72 played a crucial role in the malignant progression of PCa, and could serve as a prognostic indicator and therapeutic target for patients with PCa.

## Data Availability

the datasets used and analysed during the current study are available from the corresponding author on reasonable request.
